# Subjective experience of ‘Stream of Consciousness Impairment’ in three patients with severe amnesia

**DOI:** 10.1093/nc/niag049

**Published:** 2026-08-14

**Authors:** Anaïs Servais, Fleur Gérard, Hélène Mirabel, Marie Rafiq, Marie Wolfrum, Chloé Bost, Fabrice Bonneville, Jérémie Pariente, Emmanuel J Barbeau

**Affiliations:** Univ Toulouse, CerCo, CNRS UMR5549, CHU Purpan, Pavillon Baudot, Place du Docteur Baylac, 31300 Toulouse, France; Department of Neurology, CHU Purpan University Hospital, Place du Docteur Baylac, 31300 Toulouse, France; Department of Neurology, CHU Purpan University Hospital, Place du Docteur Baylac, 31300 Toulouse, France; Department of Neurology, CHU Purpan University Hospital, Place du Docteur Baylac, 31300 Toulouse, France; Department of Neurology, CHU Purpan University Hospital, Place du Docteur Baylac, 31300 Toulouse, France; Department of Neurology, CHU Purpan University Hospital, Place du Docteur Baylac, 31300 Toulouse, France; Department of Neurology, CHU Purpan University Hospital, Place du Docteur Baylac, 31300 Toulouse, France; Department of Neurology, CHU Purpan University Hospital, Place du Docteur Baylac, 31300 Toulouse, France; Univ Toulouse, ToNIC, UMR 1214 Inserm, Place du Docteur Baylac, 31300 Toulouse, France; Univ Toulouse, CerCo, CNRS UMR5549, CHU Purpan, Pavillon Baudot, Place du Docteur Baylac, 31300 Toulouse, France

**Keywords:** anterograde amnesia, autoimmune encephalitis, time disorientation, memory, time consciousness

## Abstract

Patients with severe anterograde amnesia have been described as ‘stuck in time’; their mental time travel abilities are altered, and they seem to live in the present. Here, we examine an unusual phenomenon in which severely amnesic patients recurrently report the belief that they have just awakened from an unconscious state. A famous case illustrating this phenomenon was that of Clive Wearing who repeatedly wrote ‘*I am now awake*’ in his diary. This unique cognitive phenomenon has not been specifically studied until now. We report three new patients who described similar subjective experiences. All three patients suffered from autoimmune encephalitis and showed massive bilateral hippocampal inflammation causing severe anterograde amnesia. We propose to call this phenomenon ‘*Stream of Consciousness Impairment*’, a reference to William James’s theory according to which, normally, we experience an uninterrupted flow of consciousness. This condition should be added to the spectrum of neurological symptoms that contribute to the study of memory and consciousness in humans.

## Teaser

- We document a specific subjective experience reported by three patients with autoimmune encephalitis.
- This subjective experience involves brief episodes of sudden awareness awakening.
- Patients have massive damage to the hippocampus and severe amnesia.
- Severe amnesia may involve disrupted continuity in the stream of consciousness.
- We propose to refer to this experience as “Stream of Consciousness Impairment’.

## Introduction

Temporal orientation is a key aspect of human cognition. It is therefore assessed in most neurological consultations. The most commonly used tests, such as the Mini Mental State Evaluation (MMSE), the Montreal Cognitive Assessment, and the Orientation subscale of the Weschler Memory Scale include a brief screening of time orientation, e.g. by asking about the date. The severity of the error (an error of 1–2 days may be considered minor, whereas an error of months or years may signal confusion or severe time disorientation), if present, may alert the clinician and warrant further investigation.

Patients with memory impairment, such as those with Alzheimer’s disease or Korsakoff’s syndrome, typically have difficulty answering such questions because they appear to be lost in time. Patients with severe anterograde amnesia have been described as ‘stuck in time’ (Tulving 2002). Their chronesthesia, i.e. their ability to have a constant awareness of the past and the future, is altered (Tulving 2002). Specifically, amnesic patients seem to show impaired mental time travel when trying to remember the episodic past or project themselves into the future (Andelman et al. 2010), which has been described as an alteration of temporal consciousness (i.e. a specific form of consciousness that enables individuals to experience their personal past, be oriented in their present, and anticipate their personal future; Dalla Barba and La Corte 2013). As a consequence, amnesic patients with hippocampal damage tend to think more about the present, while healthy subjects think more about either the past or the future (McCormick et al. 2018).

However, the clinical manifestations of temporal disorientation can vary significantly depending on the nature and extent of the memory impairment. While amnesic patients often exhibit deficits in episodic memory and mental time travel, their semantic understanding of time and its consequences may remain intact (Hoerl 1999; Craver et al. 2014). For instance, Craver et al. (2014) reported that patient K.C., despite profound anterograde amnesia and an inability to imagine episodic future events, could still reason about the long-term consequences of his decisions (Craver et al. 2014). This challenges the notion that amnesic patients are simply ‘stuck in time’ and highlights that temporal orientation depends on multiple cognitive subprocesses, which can be selectively disrupted by brain lesions.

Here, we examine an unusual phenomenon in which patients recurrently report the belief that they have just awakened from an unconscious state as if they experience a temporary alteration of their sense of passage of time. A famous case illustrating this is that of Clive Wearing, whose herpetic encephalitis caused one of the most severe cases of amnesia ever reported (Wilson and Wearing 1995). His story has been told in several documentaries, such as ‘The Man with the 7 Second Memory’ by Jane Treays in 2005, showing his peculiar relationship with time. Undoubtedly the most remarkable fact was his habit of repeatedly writing ‘*I am now awake, I do live*’ in his diary, showing his frequent belief, minute by minute, that he had just awoken from an unconscious state. He would also write ‘*I am alive now, this illness has been like death till now*’, ‘*The first time for... years... months...*’ or even ‘*Eyesight works completely again now*’. Here, we want to emphasize this particular moment of subjective realisation. It seems to refer to a specific moment when the patient suddenly felt he had become conscious ‘for the first time’. In this case, time disorientation was not just a matter of living in the present, but rather of having occasional bursts of presence in the present.

Although Clive Wearing is a famous case, this specific cognitive experience, which is not present in all types of amnesia, has not been specifically reported, as far as we know. We have recently observed three similar cases, suggesting that this very peculiar subjective experience may be more common than so far reported. We provide videos of each patient. Such cognitive phenomenon may be clinically significant, as the patients we describe below reported similar experiences. Even more importantly, this specific experience may help studying the concept of ‘Stream of Consciousness’, in reference to William James's (1890) concept of consciousness as a continuous stream that contains no break, rupture, or division.

## Case reports

In the Neurology unit of the Toulouse University Hospital, we observed three cases of patients who repeatedly described similar experiences to those of Clive Wearing. Patient 1 reported e.g. that ‘*I have a fairly surreal feeling... I now realise that I am in this room, without being aware that I was already here before. It feels like I just woke up and all my perceptions feel immediate and novel.*’ (video 1). Patient 2 said ‘*I feel that I’m lost. I can’t believe what’s happening to me. This is the first time this has happened to me, it’s weird. I no longer know the time, as if I was here without being here.*’ (video 2), or again ‘*I have the impression that I just woke up from a three-minute nap*’. Likewise, Patient 3 said ‘*I feel like I’m re-existing today, I feel like I haven’t seen or heard from myself in forever, as if you had given me a sleeping cure. Right now, I feel like I haven’t lived for a while.*’ (video 3). This specific subjective experience was observed during clinical interviews with the patients and seemed to occur in brief moments during which the patients expressed their puzzlement. The full transcriptions of the videos of the clinical interviews translated into English are available in the Supplementary Materials.

The patients were part of the ‘EXPLAINEUR’ cohort including cases of rare neurological disorders with autoimmune origin (ClinicalTrials.gov ID: NCT04698421). The ethics requirement for this specific study was satisfied by approval from the Toulouse University Hospital (2024–5; see Supplementary Material Supp. 1 the Ethics Approval section below for details).

**Figure 1:**
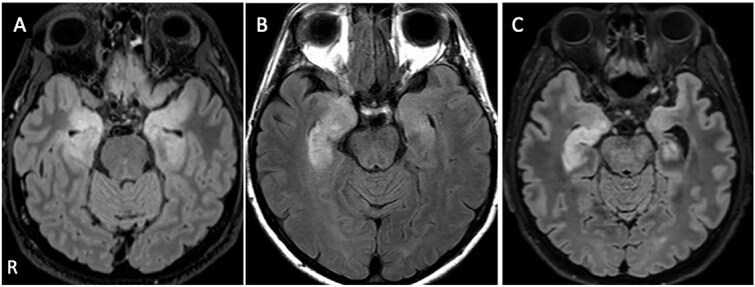
Axial FLAIR images at onset for the three patients revealing hyperintensities in the hippocampus and limbic system. (A) On the left, the axial FLAIR image of Patient 1 shows abnormal hyperintensity of both amygdala-hippocampal complexes, extending to the left basifrontal cortex and left insula. (B) In the middle, the axial FLAIR image of Patient 2 shows asymmetrical hyperintensity in both amygdala-hippocampal complexes, predominant on the right side, with atrophy of the left hippocampus. (C) On the fight, the axial FLAIR image of Patient 3 shows bilateral hyperintensity of amygdala-hippocampal complexes, predominant on the right side. R: right. Other images as well as follow-ups are presented in Supplementary Material 1.

Interestingly, all three patients were suffering from autoimmune encephalitis, albeit related to different antibodies. All of them had massive and relatively isolated bilateral brain damage to the hippocampus (see Fig. 1 for a summary, and Supplementary Figures 1, 2, and  3 for the detailed brain imaging of each patient respectively) causing severe anterograde amnesia (see Table 1 for memory assessment and Supplementary Table 1 more extensive cognitive assessment). The case descriptions for the three patients are provided below.

**Table 1:** Neurological and neuropsychological characteristics. MMSE: Mini Mental State Evaluation screened for global cognitive impairment. FCSRT: Free and Cued Selective Reminding Test (verbal memory) assessed verbal episodic memory. DMS-48: a test of visual recognition memory. AMI: autobiographical memory interview (episodic part). TEMPau: an extensive test of autobiographical memory (episodic score). Scores are interpreted as lower than the normative population of reference if Z-scores < −1.65 or percentile value <P5

		Patient 1	Patient 2	Patient 3
**Neurological history**	**Age at diagnosis**	50 years old	45 years old	54 years old
	**Gender**	M	M	F
	**Diagnosis**	Autoimmune encephalitis with anti-AK5 antibodies.	Paraneoplastic encephalitis with anti-HU antibodies, possibly favoured by an immunotherapy treatment.	Autoimmune encephalitis with anti-GAD antibodies.
**Neuropsychological evaluation**	**Delay between diagnosis and evaluation**	116 days	20 days	67 days
	**MMSE**	Total: 21/30 Orientation in space 2/5 and time 3/5	Total: 20/30 Orientation in space 3/5 and time 0/5	Total: 21/30 Orientation in space 4/5 and time 1/5
	**Verbal episodic memory**	The FCSRT was interrupted after significant difficulties on the first two free recalls. Percentile < 1.	FCSRT not administered due to the severity of the amnesia. Instead, a simpler 5-word test was administered. Results: 3/10.	FCSRT The three free recalls were severely impaired: *Z-scores* = −3.80; −4.34; −4.98 Percentile to the total cued differed recall was < 1.
	**Visual episodic memory**	Recall of Rey Figure *Z-scores* = −4.49	Recall of Rey Figure *Z-scores* = −4.49	Recall of Rey Figure *Z-scores* = −4.49
		DMS-48 Set 1 *Z-scores* = −23.5; Set 2 *Z-scores* = −25.5	5-figure test: 8/10	DMS-48 Set 1 *Z-scores* = −19.3
	**Autobio-** **graphical memory**	AMI Childhood: <P5 Young adult: <P5 Recent period: <P5	Not administered.	TEMPau (*Z-scores*) 0–17 yo: −3.8 18–30 yo: −3.5 30+ yo: −4.88 Last 5 years: −4.54 Recent: −7.97

### Patient 1

#### Medical history

Patient 1 was a 50-year-old man at the time of diagnosis. His medical history included arterial hypertension and gout, with no reported prior neurological or psychiatric history. The first symptoms appeared in June 2019, when his sister noticed behavioural changes, mainly characterized by increased emotionality. In July 2019, he developed marked fatigue and spatial disorientation, repeatedly getting lost. Difficulties also emerged at work, where he required more frequent checking and reminders. The clinical picture appeared to worsen rather abruptly in August 2019, with the onset of prominent memory impairment and temporo-spatial disorientation. In August 2019, he travelled with his family, and his memory difficulties had clearly worsened by the time he returned. He was referred to the emergency department in September 2019.

#### Clinical presentation and neurological examination

On admission, the patient showed psychomotor slowing and marked disorientation in time and space. Neurological examination did not reveal any motor or sensory deficit, cranial nerve abnormality, visual impairment, cerebellar syndrome, language disorder, agnosia, or apraxia. By contrast, he presented with a severe anterograde amnesia, together with retrograde autobiographical memory impairment. For example, he was unable to give the age of his sons, his own address, or describe specific personal memories. There were no reported episodes of unresponsiveness, delirium, hallucinations, delusions, or seizure-like events during the clinical assessment.

#### Neuroimaging and complementary investigations

The initial lumbar puncture revealed lymphocytic meningitis with elevated cerebrospinal fluid protein levels. Electroencephalograms did not show epileptic abnormalities. Brain MRI showed bilateral FLAIR hyperintensities of the amygdala-hippocampal complexes, extending to the left basifrontal cortex and left insula. The final diagnosis was autoimmune encephalitis associated with anti-AK5 antibodies.

#### Neuropsychological assessment

The clinical profile was dominated by a massive anterograde memory deficit, associated with substantial impairment of autobiographical retrograde memory and temporo-spatial disorientation. In contrast, language, praxis, gnosis, and elementary neurological functions were preserved on clinical examination. Results to the memory tests are presented in Table 1, and more extensive cognitive assessment is presented in Supplementary Table 1.

#### Clinical evolution

The patient was treated with cyclophosphamide and rituximab, initially combined with intravenous corticosteroids and intravenous immunoglobulins. Severe amnesia persisted at the follow-up memory evaluation performed at month nine (see Supplementary Table 2). There was no clinical or biological sign of neurodegenerative pathology.

### Patient 2

#### Medical history

Patient 2 was a 45-year-old man at the time of diagnosis. His medical history was notable for small-cell lung carcinoma with mediastinal and bone metastases, diagnosed in summer 2022. He had been treated with cisplatin, etoposide, and durvalumab from August to October 2022, followed by durvalumab alone. He had also received mediastinal radiotherapy and prophylactic whole-brain radiotherapy. In July 2023, he developed an anterograde memory disorder that led him to attend the emergency department.

#### Clinical presentation and neurological examination

On admission, neurological examination revealed no motor or sensory deficit and no gait instability. There was no oculomotor disorder or cranial nerve involvement. Deep tendon reflexes were present, and there was no pyramidal syndrome. The patient presented with a very severe anterograde amnesia, with forgetting occurring within a few seconds to a few minutes. There was no naming impairment and no evidence of apraxia or visual agnosia. The patient reported tinnitus in the right ear, and there were no sign of unresponsiveness, delirium, hallucinations, delusions, or seizure-like events during the clinical examination.

#### Neuroimaging and complementary investigations

The initial lumbar puncture showed no abnormality. Electroencephalography showed right frontal slowing, without epileptic abnormalities. Brain MRI revealed asymmetrical hyperintensity in both amygdala-hippocampal complexes, predominant on the right side, with atrophy of the left hippocampus*.* The final diagnosis was anti-Hu antibody-associated paraneoplastic encephalitis, possibly favoured by immunotherapy. The clinical picture included severe memory impairment and involvement of the right auditory nerve.

#### Neuropsychological assessment

The neuropsychological profile was dominated by a massive anterograde memory impairment, with very rapid forgetting over a timescale of seconds to minutes. In contrast, visual recognition was well preserved, and language, praxis, and visual gnosis were clinically spared. Detailed results to the administered memory tests are presented in Table 1, and more extensive cognitive assessment is presented in Supplementary Table 1.

#### Clinical evolution

The patient was treated with intravenous immunoglobulins, intravenous corticosteroid boluses followed by progressive oral tapering, six courses of cyclophosphamide, and rituximab induction. He remains under treatment. At the latest available evaluation, he continued to show massive anterograde memory impairment, with relatively preserved recognition. Severe amnesia persisted at the follow-up memory evaluation performed at month six (see Supplementary Table 2). There was no clinical or biological sign of neurodegenerative pathology.

### Patient 3

#### Medical history

Patient 3 was a 54-year-old woman at the time of diagnosis. Her medical history included a stroke in 2008, responsible for a left inferior homonymous quadrantanopia, as well as a previous episode of acute anterograde amnesia of epileptic origin. In July 2010, following a discussion with family members, she developed incoherent speech and behavioural changes that progressively worsened, together with massive anterograde memory impairment. She was then admitted to the hospital.

#### Clinical presentation and neurological examination

On clinical examination in July 2010, the patient presented with massive anterograde amnesia, with marked forgetting of ongoing events and severe rapid forgetting. She also showed extremely severe retrograde amnesia. The clinical picture was associated with prominent anxiety. The available clinical information did not report additional focal neurological deficits beyond the pre-existing left inferior homonymous quadrantanopia.

#### Neuroimaging and complementary investigations

EEG recordings were occasionally abnormal at onset but resolved afterwards. Brain MRI images showed bilateral hyperintensity of amygdala-hippocampal complexes, predominant on the right side*.* The final diagnosis was autoimmune encephalitis associated with anti-GAD antibodies, responsible for severe memory impairment, partial temporal lobe epilepsy, and an anxiety-depressive syndrome.

#### Neuropsychological assessment

The neuropsychological profile was dominated by massive anterograde amnesia, associated with severe retrograde amnesia. The patient showed rapid forgetting of ongoing events. Detailed results to the administered memory tests are presented in Table 1, and more extensive cognitive assessment is presented in Supplementary Table 1.

#### Clinical evolution

The patient was treated with intravenous immunoglobulins, 22 courses of cyclophosphamide, and rituximab. Severe amnesia persisted at the follow-up memory evaluation performed at month six (see Supplementary Table 2). The clinical picture also remained marked by anxiety. At the last available reassessment, in June 2023, she continued to present with prominent anterograde amnesia. There was no clinical or biological sign of neurodegenerative pathology.

## Discussion

### Phenomenology of the episodes

Patient Clive Wearing and Patients 1, 2, and 3 reported here all exhibit a peculiar subjective experience in which they appear from time to time to ‘awaken’ and become ‘conscious’ for the first time. Two of our patients (1 and 2) found this peculiar, whereas Clive Wearing and Patient 3 did not seem to criticize this particular phenomenon. Although amnesic patients are often described as living in a permanent present tense (Corkin 2013), what the patients in this series report is a specific sensation that feels like becoming suddenly conscious again of what is currently present. It is difficult to imagine the mental life of these patients, and future work is needed to clarify this. In the general population, e.g. mind wandering accounts for more than 50% of mental life and is generally oriented towards the personal past or future (Krans et al. 2015). It is not yet known whether these patients have no or impoverished mental life, or what this feels like.

Time orientation serves as a fundamental thread that weaves self-continuity, allowing individuals to perceive the unfolding of their existence as a coherent and continuous narrative. Self-continuity (Sedikides et al. 2023), as the basis of personal identity, is likely to function differently in severely amnesic patients. This is evoked, e.g. by Patient 3, who says she has not seen herself for some time and suddenly rediscovers herself.

### Stream of Consciousness Impairment

We propose to call this phenomenon ‘Stream of Consciousness Impairment’, a term inspired by William James's (1890) concept of consciousness as a continuous stream that contains no break, rupture, or division. The observation of Stream of Consciousness Impairment raises new questions. The philosopher Husserl (1966), in his ‘Phenomenology of the Consciousness of Inner Time’, considered the synthesis of ‘inner time consciousness’ to be the lowest level of mental life. Husserl argued that the coherent integration of successive moments is essential for directing ourselves towards relevant goals, i.e. the ‘intentional arc’. According to him, this intentional arc emerges from the integration of the sense of the just past, the present, and the coming future. Interestingly, both James and Husserl, based on theories from philosophy, considered the stream of consciousness to be a fundamental aspect of mental life. Here, we suggest that it can be specifically impaired in some neurological cases of severe amnesia.

### Implications for theories of consciousness

The observation of what we call here ‘Stream of Consciousness Impairment’ may help distinguish between the mechanisms that make information consciously accessible at a given moment and those that allow conscious experience to be experienced as a continuous stream across time. Despite profound amnesia, the patients remained awake, responsive, linguistically competent, and able to describe their current experiences. Their impairment therefore did not correspond to a global loss of consciousness (Laureys 2005).

This dissociation is particularly relevant to the Memory Theory of Consciousness (Budson et al. 2022). According to this account (Budson et al. 2022), consciousness evolved as part of the episodic-memory system and conscious experience is not a direct, continuous recording of the world. Instead, the brain constructs each conscious moment using processes closely related to those used in remembering and imagining (Budson et al. 2026). A key implication is that memory does more than preserve an experience after it occurs: memory processes help create and connect conscious experiences as they unfold. Consciousness therefore depends on integrating current sensory information with prior knowledge, recent events, and expectations. For our patients, the theory offers a possible explanation for why they remain awake, responsive, and aware of the immediate present, yet repeatedly feel that they have ‘just become conscious.’ Severe failure to retain or integrate the immediately preceding experience may prevent one conscious moment from being linked to the next, making each present moment feel like a new beginning. This would suggest an impairment of the continuity of consciousness, rather than a complete loss of consciousness.

This also aligns with the perspective developed by McComas (2022), according to which the hippocampus is a necessary substrate of consciousness as he conceives consciousness as an ever-changing sequence of short-term memories generated through hippocampal activity. From this perspective, the apparent continuity of consciousness depends on the continuous registration and linking of successive moments. The ‘Stream of Consciousness Impairment’ may therefore arise when bilateral hippocampal dysfunction disrupts this linkage: the patients remain conscious of the current moment, but that moment is insufficiently connected to the immediately preceding one and is consequently phenomenologically experienced as a renewed awakening.

Taken together, these memory-based accounts of consciousness provide a plausible framework for interpreting the ‘Stream of Consciousness Impairment’ as a disruption of the temporal continuity of experience rather than of consciousness itself. Nevertheless, the present cases should be regarded as hypothesis-generating. Profound amnesia does not appear to invariably appear to produce recurrent experiences of ‘awakening,’ suggesting that memory impairment alone may be insufficient to explain the phenomenon. However, it remains possible that the experience reported here has previously gone unnoticed, and that our report will help identify similar cases with different etiologies.

Systematic comparisons between amnesic patients with and without this experience will be necessary to clarify which mechanisms allow conscious experience to be perceived as continuous across time. Below, we discuss how the shared neuroanatomical and etiological characteristics of the present cases provide initial clues regarding the conditions under which the ‘Stream of Consciousness Impairment’ may arise.

### Neuroanatomical and etiological considerations

All three of our patients suffered from clinically severe amnesia associated with encephalitis affecting medial temporal lobe structures bilaterally. This seems to be a common pattern among the patients although this is still rather general. The diagnosis of autoimmune encephalitis has progressed rapidly in recent years, particularly with the establishment of expert centres. It is therefore possible that more cases will be reported in the near future, helping to clarify why some patients exhibit this specific subjective experience, and adding to our knowledge of this neurological condition.

The neuroanatomical lesion pattern shared by the patients—primarily affecting the bilateral hippocampi and amygdalae—may help explain the observed alterations in temporal and self-consciousness. The hippocampus is known to play a central role in encoding and integrating episodic memories within their spatial and temporal contexts (Eichenbaum 2013), contributing to the subjective experience of time and the continuity of autobiographical memory. This function could be supported by the presence of time cells discovered in the hippocampus (Eichenbaum 2014). Damage to these structures may therefore disrupt the temporal coherence of conscious experience (McComas 2022), leading to fragmented or altered perceptions of time and self.

This interpretation can be further informed by comparison with Korsakoff syndrome. Although Korsakoff syndrome is characterized by profound anterograde amnesia, disorientation, and confabulation (Arts et al. 2017), patients do not typically report recurrent spontaneous experiences of having just become conscious or of ‘re-existing’ for the first time. While hippocampal abnormalities may occur, Korsakoff syndrome is more classically associated with thiamine deficiency and damage to diencephalic–limbic circuits (Arts et al. 2017). In addition, even patients with Alzheimer’s disease, which commonly involves hippocampal atrophy, do not usually report the subjective experience of ‘Stream of Consciousness Impairment’. Interestingly, some studies report asymmetric hippocampal atrophy in Alzheimer’s disease, with the left hippocampus showing more pronounced volume reduction (Pusparani et al. 2025). These observations suggest that ‘Stream of Consciousness Impairment’ cannot be explained by left-sided hippocampal vulnerability alone, but may require a more specific disruption, potentially bilateral limbic dysfunction. A similar contrast can be drawn with limbic-predominant age-related TDP-43 encephalopathy (LATE), a common age-related proteinopathy associated with amnestic dementia and sometimes hippocampal sclerosis (Nelson et al. 2019). Unlike the acute autoimmune cases reported here, LATE is typically slowly progressive. The absence of reported ‘Stream of Consciousness Impairment’ in LATE and typical Alzheimer’s disease may therefore reflect the difference between gradual hippocampal degeneration and acute bilateral hippocampal dysfunction. While gradual deterioration can be thought to allow partial compensatory adaptation, acute disruption may abruptly interrupt the temporal scaffolding of conscious self-continuity.

Taken together, these comparisons suggest that ‘Stream of Consciousness Impairment’ is unlikely to result from hippocampal damage alone. A broader limbic–diencephalic network may be involved. This view is consistent with recent proposals that episodic memory depends not only on the hippocampus, as is traditionally proposed, but also on thalamic structures, particularly the anterior thalamic nuclei, which may contribute to memory independently of the hippocampus (Aggleton and O’Mara 2022; Aggleton et al. 2023). Supporting this idea, a recent study of 38 patients with autoimmune limbic encephalitis found consistent atrophy of these nuclei, as well as of the mammillary bodies and hippocampus. Moreover, diencephalic volumes predicted memory performance as well as hippocampal atrophy (Argyropoulos et al. 2026). As this system requires synchronization of many brain regions, a hypothesis could be that it is the transient disruption of this synchronization that leads to these episodes of subjective reawakening. This hypothesis may help explain why patients with more isolated hippocampal lesions do not typically report this symptom, whereas patients with acute, bilateral, and more widespread limbic involvement may be more vulnerable to such alterations in conscious self-continuity.

These neuroanatomical and functional considerations generate hypotheses about the links between the observed phenomenological impairments and underlying brain mechanisms, and may guide future research toward a more integrated understanding of the disruptions in consciousness associated with these lesions.

### Clinical characterization

To help neurologists identify ‘Stream of Consciousness Impairment’, we propose the following criteria: (i) a sudden, spontaneous and brief subjective feeling of waking up in the present time (whereas the subject was not sleeping), (ii) the recurrence of such episodes (iii) in the presence of retrograde autobiographical amnesia and (iv) occurring in the context of very severe anterograde amnesia without intellectual alteration. To date, we are unable to provide diagnostic tools (e.g. specific questions that could be asked to the patient to observe this phenomenon), as it remains unclear what instances trigger these sudden, spontaneous, and transient ‘awakenings’. Similarly, we cannot determine the required severity of amnesia or how to measure it precisely when it is so profound that a comprehensive neuropsychological assessment is not feasible.

However, these characteristics could help differential diagnosis with other phenomena occurring during other forms of severe amnesia. For example, transient global amnesia is also characterized by highly severe amnesia (Quinette et al. 2006), but we are not aware that these patients ever report phenomena as described here. Instead, patients with transient global amnesia seem to have repetitive questioning about where they are but not about ‘awakening’.

If this phenomenon exists in other types of amnesia, it has remained under the radar of researchers until now. For example, in Milner et al. (1968), it is noted that the famous patient H.M., whose amnesia developed after a bilateral medial temporal lobe lobectomy (including the hippocampus, entorhinal cortex, and amygdala), reported similar content of thought, but without evidence of the same phenomenological experience. We quote ‘He (meaning H.M.) often volunteers stereotyped descriptions of his own state, by saying that it is ’like waking from a dream.‘ His experience seems to be that of a person who is just becoming aware of his surroundings without fully comprehending the situation, because he does not remember what went before’ (Milner et al. 1968, page 217). Although the vocabulary used by H.M. is very close to that of our patients, the available descriptions do not make it possible to determine whether he experienced the same *sudden* and *spontaneous feeling of awakening* that we observed in our patients. In Milner et al. (1968), this was interpreted as a stereotyped description of his state (Milner et al. 1968; Hoerl 1999), whereas in our patients the phenomenon appeared as brief, spontaneous episodes, often accompanied by puzzlement and expressed in variable terms. Furthermore, we would like to explicitly mention that, in our patients, this phenomenon occurred exclusively in the absence of prior sleep, in contrast to patients with transient epileptic amnesia, who most commonly experience amnestic episodes upon awakening from sleep (Butler et al. 2021).

Intriguingly, amnestic patients, including H.M., estimate time accurately, except that they tend to underestimate the duration of longer intervals (Richards 1973). In our patients, however, time estimation, duration judgment, and more detailed aspects of temporal orientation were not systematically assessed. The available clinical data, including MMSE temporal orientation items, are therefore insufficient to determine whether Stream of Consciousness Impairment reflects an impairment of temporal orientation, duration estimation, subjective temporal continuity, episodic memory updating, or another cognitive mechanism. This raises specific questions about the pattern of lesions or network dysfunction that might account for Stream of Consciousness Impairment and its relationship with time, consciousness and memory networks. These outstanding questions need to be resolved in order to gain understanding of the cognitive processes impaired in patients with the ‘Stream of Consciousness Impairment’.

## Conclusion

Although temporal disorientation is commonly observed in disorders affecting memory, its underlying mechanisms remain complex. Overall, clinicians should be aware of this specific presentation of amnesia. It may be appropriate to add the Stream of Consciousness Impairment to the spectrum of neurological symptoms that contribute to the study of consciousness in humans. Our patients show that the continuous unfolding of conscious experience can be profoundly disrupted despite relatively preserved language, interaction, and momentary awareness. The ‘Stream of Consciousness Impairment’ may therefore help dissociate the mechanisms that enable conscious access from those that sustain conscious continuity. We argue that it is essential to go beyond the assessment of time in terms of the day and the date, to the assessment of the subjective experience of time, as this plays an important role in people’s mental lives. We believe that describing and naming this phenomenon will encourage further investigation, which will be key to understanding different profiles of amnesia.

## Supplementary Material

Supplementary_materials_niag049

## Data Availability

Videos of interviews with the patients are available as supplementary materials.
