# Supplementary material for: Subjective experience of ‘Stream of Consciousness Impairment’ in three patients with severe amnesia
Source: Neurosci Conscious. 2026 Aug 14;2026(1):niag049. doi: 10.1093/nc/niag049 (PMC13474263; doi:10.1093/nc/niag049)
Supplement: Supplementary file 1 [file supplementary_materials_niag049.zip › Supplementary_materials_niag049.docx]

**Supplementary Material**

**Supp. Table 1.** – Initial neuropsychological assessment of executive functions, praxis and language. Scores are interpreted as lower than the normative population of reference if Z-scores < -1.65 or percentile value <P5.

|  | | **Patient 1** | **Patient 2** | **Patient 3** |
| --- | --- | --- | --- | --- |
| **Neuropsychological evaluation** | **Delay between diagnosis and evaluation** | 116 days | 20 days | 67 days |
|  | **Working memory** | Span Digits  Forward  *Z-scores* = -0.92  Backward  *Z-scores* = -1.23 | Span Digits  Forward  *Z-scores* = -0.20  Backward  *Z-scores* = 0.30 | Span Digits  Forward  *Z-scores* = -0.15  Backward  *Z-scores* = -1.23 |
|  | **Planification** | Copy of Rey Figure  *Z-scores* = -0.56 | Copy of Rey Figure  *Z-scores* = -0.56 | Copy of Rey Figure  *Z-scores* = -0.55 |
|  | **Mental flexibility** | Trail Making Test  B-A (*s*)  *Z-scores* = -4.38 | Trail Making Test  B-A (*s*)  *Z-scores* = -2.62 | Trail Making Test  B-A (*s*)  *Z-scores* = -6.05 |
|  | **Inhibition** | Stroop Test  Interference - Denomination (s)  *Z-scores* = -0.82 | Stroop Test  Interference - Denomination (s)  *Z-scores* = -1.83 | Stroop Test  Interference - Denomination (s)  *Z-scores* = -0.57 |
|  | **Verbal fluency** | Phonemic (P)  *Z-scores* = -2.49  Semantic (Animal)  *Z-scores* = -1.70 | Phonemic (P)  *Z-scores* = -0.83  Semantic (Animal)  *Z-scores* = -2.40 | Phonemic (P)  *Z-scores* = -0.90  Semantic (Animal)  *Z-scores* = -2.27 |
|  | **Processing speed** | Stroop Denomination (s)  *Z-scores* = -0.10 | Stroop Denomination (s)  *Z-scores* = 0.08 | Stroop Denomination (s)  *Z-scores* = -0.66 |
|  |  | Trail Making Test (Part A)  *Z-scores* = 0.14 | Trail Making Test (Part A)  *Z-scores* = -0.07 | Trail Making Test (Part A)  *Z-scores* = 0.60 |
|  | **Language** | Not administered. | Denomination BECs  Percentile <5 | Not administered. |
|  | **Praxis** | Brief evaluation battery  (Mahieux, 2009)  5/5; 10/10; 8/8 | Brief evaluation battery  (Mahieux, 2009)  5/5; 10/10; 8/8 | Not administered. |

**Supp. Table 2.** Follow-up neuropsychological assessment of memory. Scores are interpreted as lower than the normative population of reference if Z-scores < -1.65 or percentile value <P5.

|  | | **Patient 1** | **Patient 2** | **Patient 3** |
| --- | --- | --- | --- | --- |
| **Follow-up Neuropsychological evaluation of Memory** | **Delay between diagnosis and follow-up evaluation** | 285 days | 188 days | 185 days |
|  | **MMSE** | Total: 25/30  Orientation in space 4/5 and time 2/5 | Total: 18/30  Orientation in space 2/5 and time 1/5 | Total: 23/30  Orientation in space 4/5 and time 3/5 |
|  | **Verbal episodic memory** | FCSRT  The three free recalls were severely impaired: *Z-scores* = -3.74; -3.04; -3.31  Percentile to the total cued differed recall was <1. | A 5-word test was administered and showed impairment. Results: 4/10. | A 5-word test was administered and showed impairment. Results: 6/10. |
|  | **Visual episodic memory** | Recall of Rey Figure  *Z-scores* = -2.96 | Recall of Rey Figure  *Z-scores* = -4.49 | Recall of Rey Figure  *Z-scores* = -4.49 |
|  |  | DMS-48  Set 1  *Z-scores* = -12;  Set 2  *Z-scores* = -20.5 | Not administered. | DMS-48  Set 1  *Z-scores* =-18.25 |

**Supp. Fig 1.** Patient 1. 53-year-old man with limbic encephalitis. A) and B) Axial FLAIR images at onset show abnormal hyperintensity of both amygdala-hippocampal complexes, extending to the left basifrontal cortex and left insula, as observed on C) Coronal FLAIR image. D) Axial Diffusion weighted image demonstrates no restricted diffusion, a feature in favour of auto-immune encephalitis rather than herpes simplex encephalitis. E) Post-contrast axial T1-weighted image depicts faint enhancement of the left amygdala. F) Coronal T2-weighted image at 6 months follow-up reveals severe atrophy of both hippocampi.

**Supp. Fig 2.** Patient 2, 46-year-old man with limbic encephalitis. A) Axial FLAIR image at onset reveals asymmetrical hyperintensity in both amygdala-hippocampal complexes, predominant on the right side. B) Post-contrast axial T1-weighted image depicts faint enhancement of the right amygdala. C) Coronal T2-weighted image shows swelling and hyperintensity of the right hippocampus, while there is already atrophy of the left hippocampus. D) Coronal T2-weighted image at only 2 months follow-up demonstrates slight volume loss and almost normal signal intensity of the right hippocampus, while left hippocampus remains unchanged.

**Supp. Fig 3.** Patient 3, 58-year-old woman with limbic encephalitis. A) and B) Axial FLAIR images at onset reveal bilateral hyperintensity of amygdala-hippocampal complexes, predominant on the right side. C) At 6 months follow-up, C) axial FLAIR and D) axial T2-weighted image show rapid atrophy of both hippocampi, with a persistent abnormal hyperintensity of both amygdala-hippocampal complexes.

**Video transcription of Interview of patient 1 by Prof. Jérémie Pariente**

Part 1—Illustration of his severe amnesia

- Patient 1: So, you are explaining to me that I have been here for 4 days.
- Dr.: 5 days.
- Patient 1: 5 days? and that it is September 28^th^… So, there, I have a gap in my head because I am located at the beginning of September. That’s my feeling. Have we already spoken to each other?
- Dr.: We have spoken every day since you arrived.
- Patient 1: For how long have I been here? I make you repeat, sorry doctor.
- Dr.: 5 days.

Part 2—Illustration of his Stream of Consciousness Impairment

- Dr.: According to you, how long have you been here, sir?
- Patient 1: I have no idea.
- Dr.: So I am telling you, today is the 5^th^ day.
- Patient 1: Okay.
- Dr.: The 5^th^ day of hospitalization.
- Patient 1: Where are we?
- Dr.: Toulouse Purpan Hospital, 6^th^ floor.
- Patient 1: Okay.
- Dr.: And today is Saturday, September 28. Is that good for you?
- Patient 1: In fact, my friends organized a prank…
- Dr.: No, no, do you have the impression of that?
- Patient 1: Uh, it’s quite strange, I have a rather surreal feeling, yes.
- Dr.: What is your feeling sir?
- Patient 1: Well, in fact I find myself in this room, without being aware that I was there yesterday for example. It is like if I just woken up. All the perceptions are really immediate and novel. I have no feedback…
- Dr.: Of what happened yesterday or the day before?
- Patient1: Exactly.

**Video transcription of Interview of patient 2 by Prof. Jérémie Pariente**

Part 1—Illustration of his severe amnesia

- Dr.: Could you tell me what your room number is?
- Patient 2: No, not at all.
- Dr.: So, I’ll tell you, it’s room 626, okay?
- Patient 2: Okay.
- Dr.: And it is 11:04 am.
- Patient 2: Okay.
- Dr.: And it is Saturday, July 22.
- Patient 2: Okay.
- Dr.: What’s your room number?
- Patient 2: I don’t remember.
- Dr.: I will tell you. It is room 626. And what time is it?
- Patient 2: I don’t remember.
- Dr.: 11:05 am now. Okay?
- Patient 2: Okay.
- Dr.: Repeat it.
- Patient 2: 11:05 am.
- Dr.: And could you tell me the date of the day?
- Patient 2: Neither.
- Dr.: July 22.
- Patient 2: July 2022. Okay.
- Dr.: Is that fine with you?
- Patient 2: Yes, yes, yes.
- Dr.: It is the truth.
- Patient 2: I believe you, I believe you. It is just that I feel like I am lost.
- Dr.: And your room number?
- Patient 2: I don’t remember.
- Dr.: And the time?
- Patient 2: I don’t remember either.

Part 2—Illustration of his Stream of Consciousness Impairment

- Patient 2: I don’t realize what’s happening to me. Where does it come from?
- Dr.: We are searching.
- Patient 2: Okay. This is the first time it’s happening to me. It’s weird.
- Dr.: Is has never happened before, this memory disorder?
- Patient 2: No, never. It’s weird.
- Dr.: What is bizarre? Tell me a little bit what is this feeling?
- Patient 2: It is like being lost. We don’t know the time anymore. We don’t know all of this anymore. Like we were here, but without being there. It feels weird.
- Dr.: Does it make you anxious?
- Patient 2: Yes.

**Video transcription of Interview of patient 3 by her neuropsychologist**

Part 1—Illustration of her severe amnesia

- Dr.: Did you eat today?
- Patient 3: I don’t know.
- Dr.: You don’t know.
- Patient 3: I don’t know, I’ll assume so.
- Dr.: What did you eat?
- Patient 3: I don’t know.
- Dr.: You don’t know. It’s about 2PM, so obviously you ate. It was not very long ago that you ate. You don’t remember at all what was for lunch?
- Patient 3: No, no.
- Dr.: The hospital kitchen did not strike you, right?
- Patient 3: No, no.
- Dr.: Were there green beans?
- Patient 3: I don’t know at all.
- Dr.: Some pasta?
- Patient 3: I don’t know.
- Dr.: Semolina?
- Patient 3: That does not ring a bell.
- Dr.: Neither one not the other?
- Patient 3: No, no.
- Dr.: Probably you are couscous.
- Patient 3: I don’t know.

Part 2—Illustration of her Stream of Consciousness Impairment

- Dr.: Do you have more recent memories? Some event closer to the current period?
- Patient 3: Uh, no. No, not so much.
- Dr.: It would be only very old memories that you would have kept?
- Patient 3: Yes, rather.
- Dr.: Okay, and do you have memories of your personality? What kind of person are you?
- Patient 3: A hyperactive person. But it is the first… I feel like I am re-existing today, I feel like I haven’t seen or heard from myself in forever, as if you had given me a sleeping cure. Right, now, I feel like I haven’t lived for a while.
- Dr.: Do you have the impression of discovering yourself every day?
- Patient 3: Not every day, now, at this moment. I have the impression that I have not lived for a certain amount of time.
